# Imaging features of noncalcified DCIS

**DOI:** 10.1186/bcr3292

**Published:** 2012-11-09

**Authors:** JM Scudder, J Parikh

**Affiliations:** 1 Guy's and St Thomas' NHS Foundation Trust, London, UK

## Objective

To illustrate the radiological features of noncalcified DCIS (NC-DCIS) on mammography, ultrasound and MRI. To highlight the role of MRI in determining extent of disease.

## Introduction

DCIS accounts for 20% screen-detected cancers and 5% of symptomatic cancers. Eighty to 90% present mammographically as microcalcifications, but 10 to 20% are noncalcified and can be mammographically occult. With reported re-excision rates as high as 65% for breast-conserving surgery in DCIS, accurately determining disease extent on preoperative imaging is important.

## Methods

Imaging of 117 patients with pure DCIS from 2007 to 2011 was reviewed retrospectively. Fifteen patients with NC-DCIS were identified. Imaging findings were compared with disease extent on postoperative histology.

## Results

NC-DCIS appeared as follows. Mammography: occult 33%, diffuse increased breast density 33%, focal architectural distortion 13%, well-circumscribed lesion 13%, tubular ductal density 7%. Ultrasound: occult 7%, intraductal lesion 13%, microcystic lesion 13%, solid lesion 53%, ill-defined echo poor focus 13%. MRI: nonmass-like nodular enhancement in a ductal, segmental or regional distribution 83%, amorphous nonmass-like enhancement 17%. MRI best depicted the true extent of disease.

## Conclusion

Recognition of these imaging features is important for accurate surgical planning. MRI has an important role in accurately delineating disease extent and should be considered in treatment planning for NC-DCIS.
